# Supplementary material for: Squash enables Spindle-E-dependent heterotypic piRNA amplification in the Drosophila ovary
Source: iScience. 2026 Jul 20;29(8):116920. doi: 10.1016/j.isci.2026.116920 (PMC13523842; doi:10.1016/j.isci.2026.116920)
Supplement: Supplementary file 1 [file mmc1.pdf]

iScience, Volume *29*

Supplemental information

Squash enables Spindle-E-dependent heterotypic piRNA amplification in the *Drosophila* ovary

Xin Xu, Taichiro Iki, Shinichi Kawaguchi, and Toshie Kai

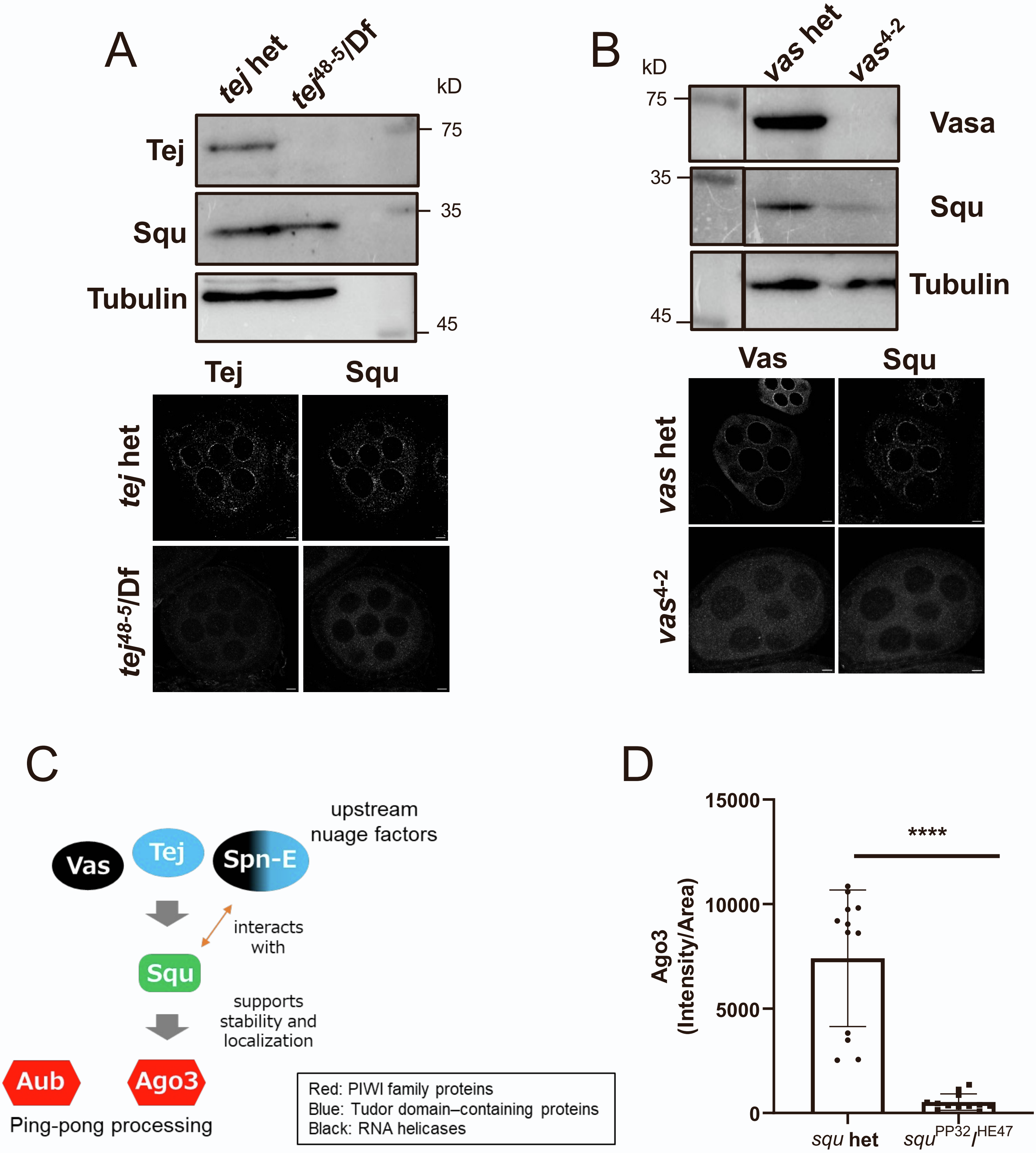

Figure S1: Subcellular localization and expression of Squ in *tej* and *vas* mutants

(A–B) Protein levels and immunostaining of Squ in control ovaries and in *tej* (A) or *vas* (B) mutant egg chambers. Scale bar is 5 µm. Molecular weight markers were obtained from the same membrane but from a region separate from the sample lanes. The marker image was placed adjacent to the blot image for reference, and the boundary is indicated by a vertical line. (C) The model for the schematic role of Squ. During the germline piRNA biogenesis, Vas, Tej and Spn-E function as the core components for the precursor processing which is essential for the initiation for ping-pong amplification. Localization of Squ depends on these core factors. Ago3, but not Aub is affected by Squ function. PIWI family proteins are colored red, Tudor domain–containing proteins are colored blue, and RNA helicases are indicated by circles. (D) Quantification of the signal intensity of the Ago3 protein around the nuage. Error bars indicate standard deviation (12 nurse cells from 3 biological replicates). Statistics: unpaired t-test, ****$p < 0.0001$.

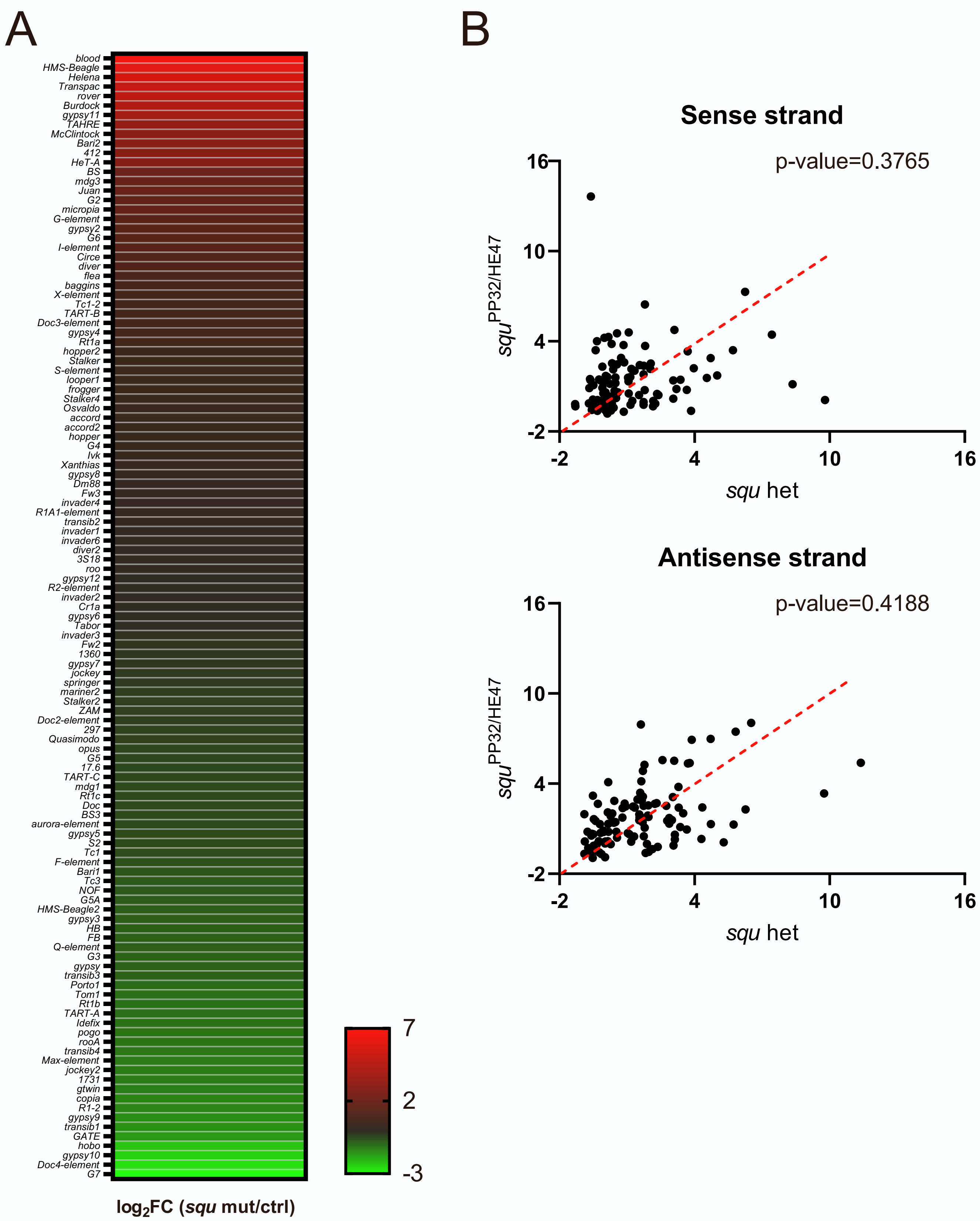

Figure S2: Transposon expression and Piwi-bound piRNA analysis in *squ* mutant ovaries

(A) The heatmap of fold changes of TEs' expression for all 119 canonical transposons in control and *squ* mutant ovaries.

(B) Z0 scores of germline Piwi-bound small RNAs mapped to 121 canonical transposons were compared between the control and *squ* mutant. Each dot represents a single TE. Both sense and antisense strands were analyzed. Statistics: paired t-test.

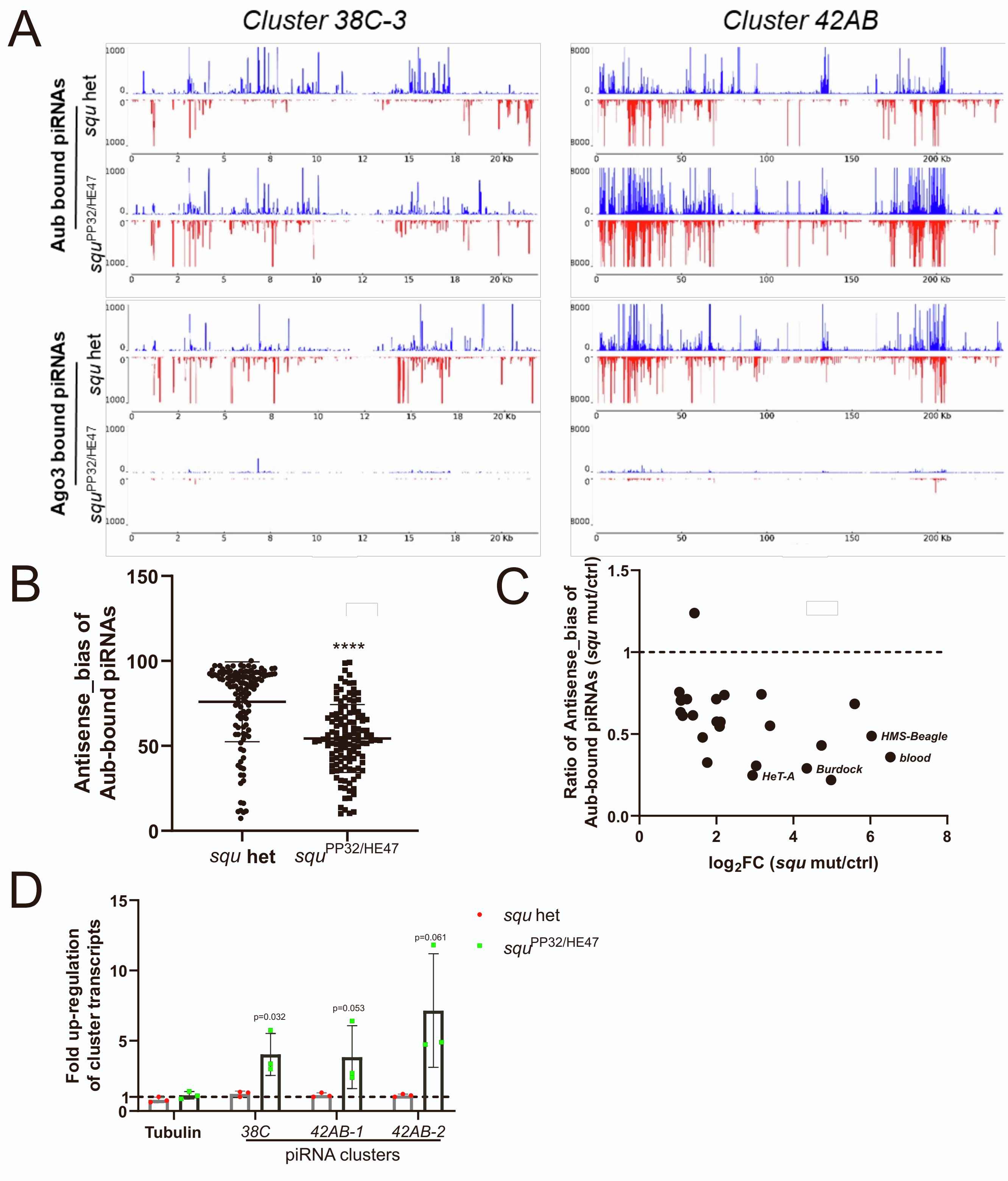

Figure S3: Second replicate for Aub- and Ago3- bound piRNAs in the control and *squ* mutant

(A) Aub- (upper panels) and Ago3-bound (lower panels) small RNAs from control and *squ* mutan ovaries mapped to the major piRNA clusters *38C-3* and *42AB* (replicate 2). Plus-strand (blue) and minus-strand (red) piRNAs are shown as upward and downward peaks, respectively. (B) Antisense bias of Aub-bound piRNAs mapping to transposable elements (TEs) in control (*squ* het) and *squ* mutant ovaries (replicate 2). Each dot represents one TE; antisense piRNA ratios are calculated by dividing antisense-mapping piRNA reads by total mapping reads for each TE. Horizontal lines indicate the mean values. ****$p < 0.0001$ (unpaired t-test). (C) Effect of loss of *squ* function on antisense ratio of Aub-bound piRNAs mapping to transposon consensus sequences. Dots indicate 24 up-regulated TEs (Fig. 2A) in *squ* mutant relative to control ovaries (replicate 2). For each TE, the antisense bias in *squ* mutant is divided by that in *squ* heterozygous. (D) RT–qPCR analysis of transcripts derived from major germline piRNA clusters *38C* and *42AB*, with tubulin as a control. All values are normalized to *rp49* and are shown relative to expression levels in the presence of *squ* heterozygous. Error bars indicate standard deviation (3 biological replicates). Statistical significance is assessed by Student's unpaired t-test.

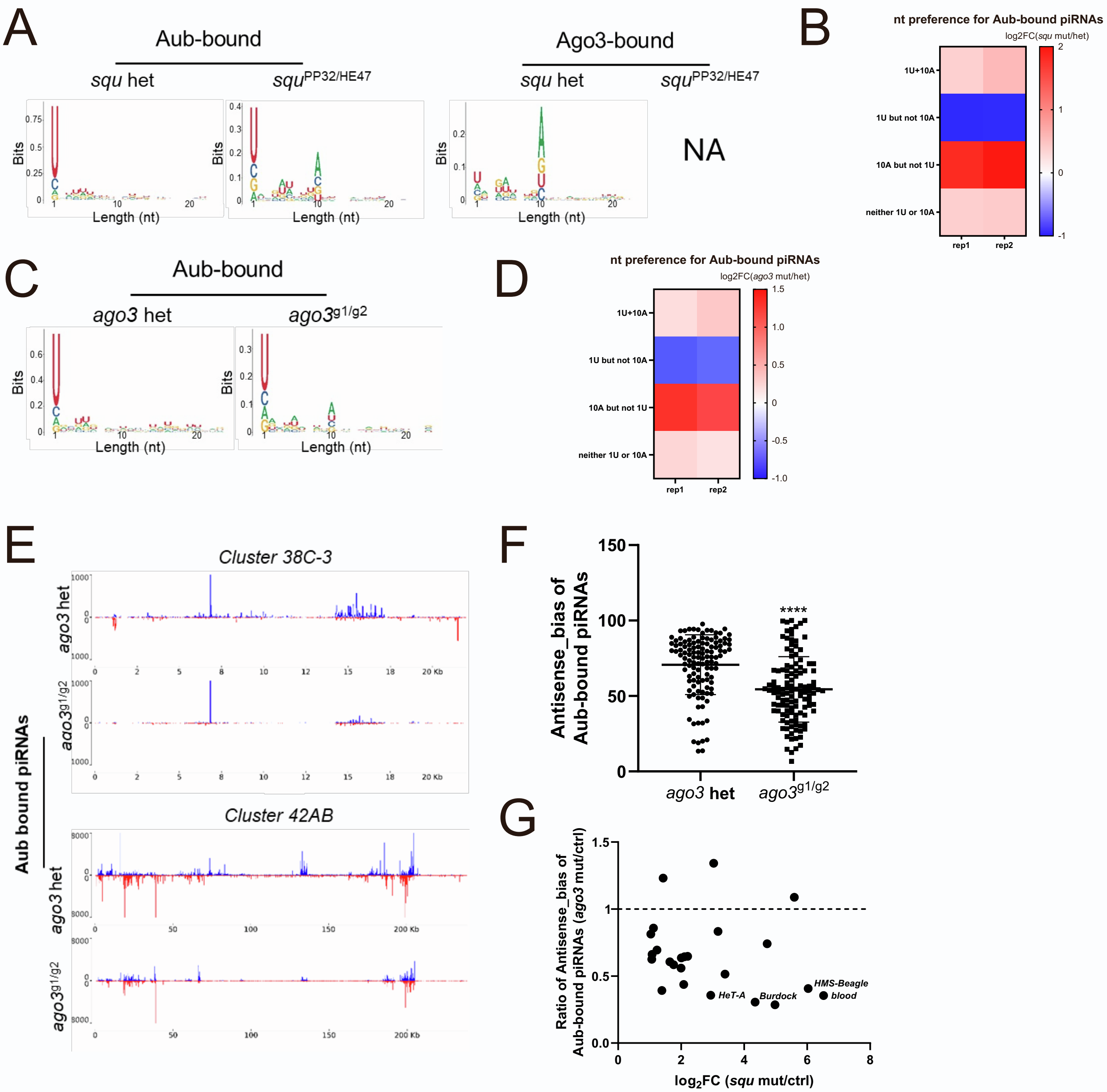

Figure S4: Second replicate for Aub-Aub homotypic signature in *squ* and *ago3* mutants

(A) Nucleotide bias of Aub-bound and Ago3-bound piRNAs mapped to piRNA cluster loci in control and *squ* mutant ovaries (replicate 2). Sequence logos indicate 1st to 20th of piRNAs. 10A signature of *squ* mutant condition reflects enhanced Aub–Aub homotypic ping-pong. NA (Not analyzed); Ago3-bound piRNAs are not detectable in *squ* mutant. (B) The log_2FC of co-occurrence analysis for the 1U and 10A biases of Aub-bound piRNAs in control and *squ* mutant ovaries (2 replicates). (C) Nucleotide bias of Aub-bound piRNAs in control and *ago3* mutant ovaries (replicate 2). (D) The log_2FC of co-occurrence analysis for the 1U and 10A biases of Aub-bound piRNAs in control and *ago3* mutant ovaries (2 replicates). (E) Aub-bound piRNAs from control and *ago3* mutant ovaries mapped to the major germline piRNA clusters *38C-3* and *42AB*. Blue; genomic plus-strand reads, red; minus-strand reads. (F) Antisense bias of Aub-bound piRNAs mapping to transposable elements (TEs) in control and *ago3* mutant ovaries (replicate 2). Each dot represents one TE; antisense piRNA ratios are calculated by dividing antisense-mapping piRNA reads by total mapping reads for each TE. Horizontal lines indicate the mean values. ****$p < 0.0001$ (unpaired t-test). (G) Ratio of antisense bias of Aub-bound piRNAs for the 24 up-regulated TEs (detected in *squ* mutant) in *ago3* mutant versus control ovaries (replicate 2). Dots indicate 24 up-regulated TEs (Fig. 2A) in *ago3* mutant relative to control ovaries (replicate 2). For each TE, the antisense bias in *ago3* mutant is divided by that in *ago3* heterozygous.

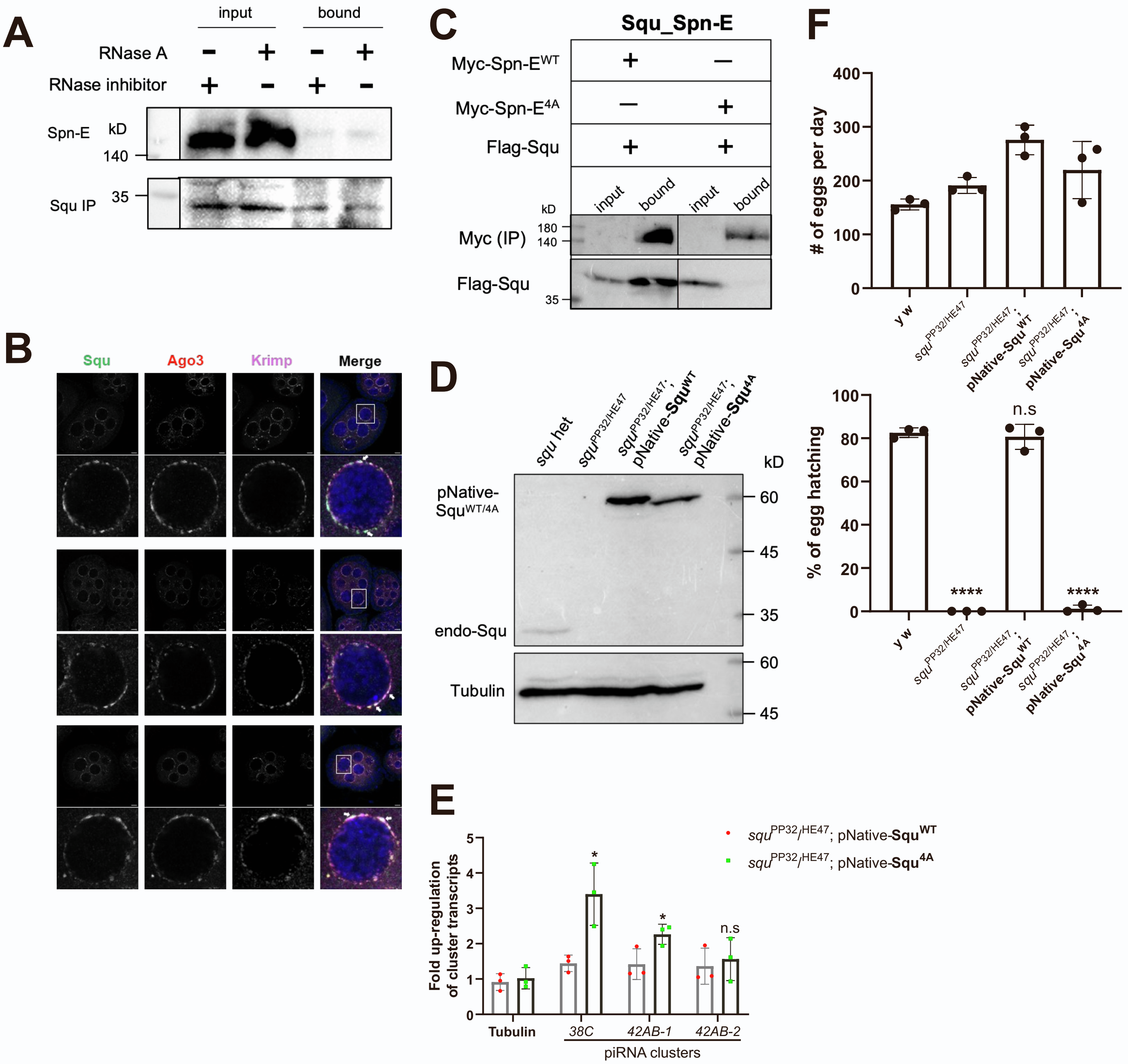

Figure S5: Interaction of Squ and Spn-E is crucial for *Drosophila* female fertility

(A) RNA dependency assay for the interaction between Squ and Spn-E from ovary lysates. Co-IP experiment were performed in the presence of an RNase inhibitor or after the RNase A treatment. (B) Localization of Squ, Ago3 and Krimp in the *squ* heterozygous control germline cells. (upper panels: stage 4–5 egg chambers; lower panels: magnified nuclei indicated by squares in the upper panels). Scale bar is 5 µm. (C) Immunoprecipitation of Myc-tagged Spn-E^{WT} and Spn-E^{4A} using anti-Myc antibodies with the co-expression of Flag-Squ in S2 culture cells. Flag-Squ can be detected in Spn-E^{WT} but not in Spn-E^{4A} immunoprecipitates. (D) Immunoblotting of proteins extracted from the heterozygous control, *squ*$^{PP32/HE47}$ and *squ*$^{PP32/HE47}$expressing Squ^{WT} or Squ^{4A} ovaries. (E) RT–qPCR analysis of transcripts derived from major germline piRNA clusters *38C* and *42AB*, with tubulin as a control. All values are normalized to *rp49* and are shown relative to expression levels in *squ*$^{PP32/HE47}$; pNative-Squ^{WT} ovaries. Error bars indicate standard deviation (3 biological replicates). Statistical significance is assessed by Student's unpaired t-test. *p <0.05. (F) Analysis of egg laying and hatching rate. Infertility of *squ* mutant is rescued by the pNative-promoter-Squ^{WT} but not Squ^{4A} mutant. Daily egg production by five females is recorded (3 biological replicates). T-test is performed for the statistics analysis. (****p <0.0001).

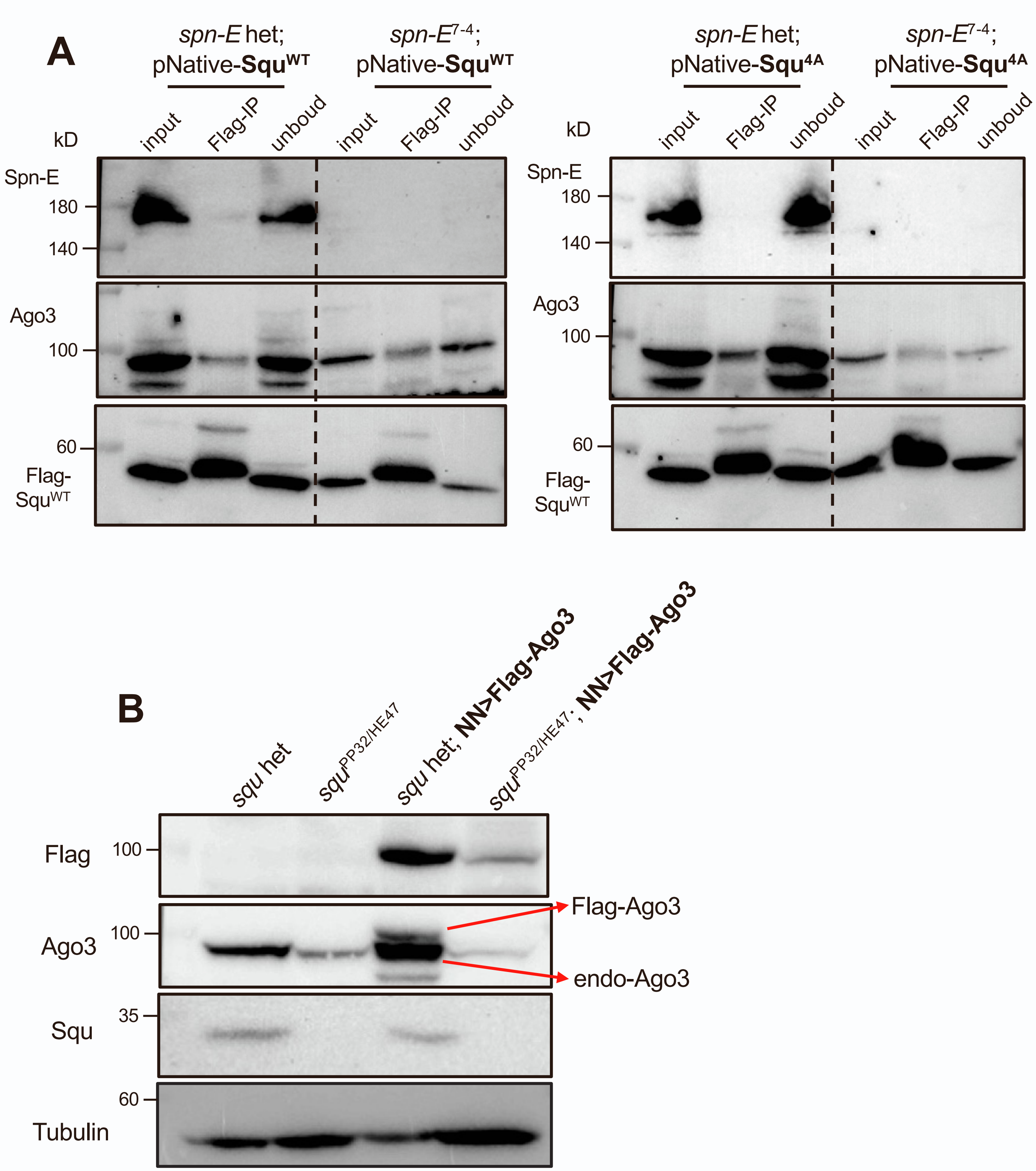

Figure S6: Squ contributes to Ago3 maintenance independently of Spn-E

(A) Immunoprecipitation of GFP–3×Flag–Squ and the variants expressed under the native promoter (pNative-Squ^{WT} and pNative-Squ^{4A}) using anti-3FLAG antibodies in the ovaries expressing pNative-Squ^{WT} and - Squ^{4A} under the loss of endogenous *spn-E* condition.

(B) Immunoblotting of proteins extracted from the heterozygous control, *squ*$^{PP32/HE47}$, *squ* heterozygous and *squ*$^{PP32/HE47}$ expressing NGT40; nosGal4 driven Flag-Ago3 protein. Two bands are detected using anti-Ago3 antibodies (upper bands are the NGT40; nosGal4 driven Flag-Ago3 and lower bands are the endogenous Ago3).

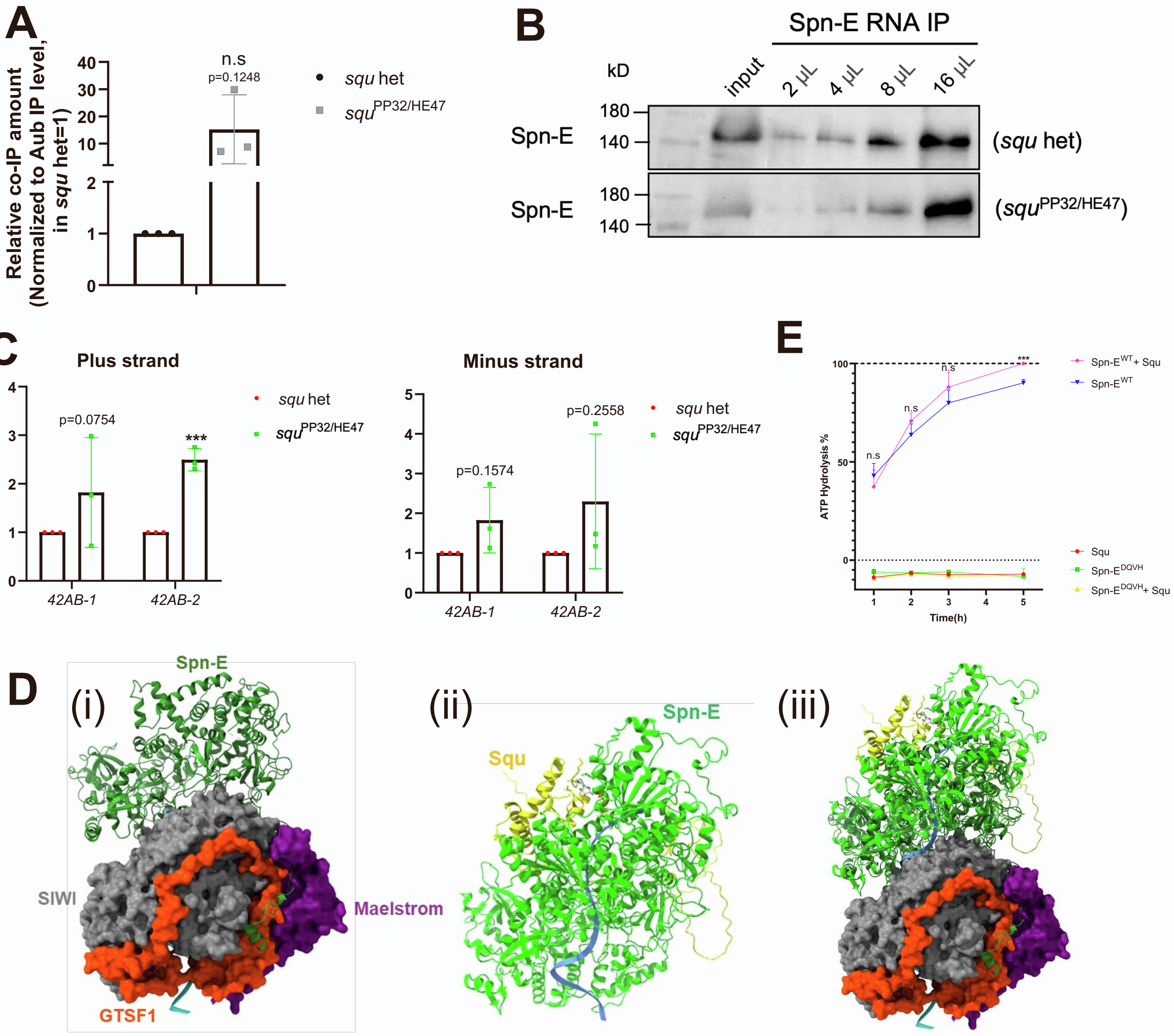

Figure S7: Structural modeling and in vitro characterization of Spn-E and Squ

(A) Quantification of intensity ratio for Aub–Spn-E interaction in the presence and absence of *squ*. Relative values are obtained by $^{\Delta\Delta}$Ct using *rp49* as reference. The signal is further normalized by the Aub protein levels in IP fractions. Error bars indicate standard deviation (3 biological replicates). The beads only controls are also shown as a negative control. Statistics; unpaired t-test. (B) Immunoblotting of the input and Spn-E IP fraction samples in the presence and absence of *squ*. 2, 4, 8 and 16 µl of the IP samples are loaded to perform the comparison of IP efficiency. (C) Strand-specific qPCR analysis for the expression of both strands of Spn-E bound cluster transcripts from *42AB* in control and *squ* mutants. Error bars indicate standard deviation (3 biological replicates). Statistics; unpaired t-test (***p <0.001). (D) Predicted structure of the SIWI*/Aub* complex with Spn-E and Squ. (i) Cryo-EM structure of the *Bombyx mori* SIWI/Aub–piRNA–target RNA–GTSF1–Maelstrom–Spn-E complex (PDB: 9NHS). SIWI is shown in gray, GTSF1 in orange, Maelstrom in purple, and Spn-E in dark green. The target RNA is depicted as a cyan ribbon. (ii) Predicted *Drosophila melanogaster* Spn-E–Squ complex generated using AlphaFold3. Spn-E is shown in green and Squ in yellow. A single-stranded RNA is displayed as a blue ribbon, and ATP is shown in stick representation. (iii) Structural superposition of the two models based on alignment of their Spn-E subunits. (E) ATPase activity of purified Spn-E^{WT}, Spn-E^{DQVH}, and Squ. ATP hydrolysis is measured up to 5 h, and released inorganic phosphate is quantified. Spn-E^{WT} shows robust ATPase activity, whereas Spn-E^{DQVH} and Squ alone exhibit minimal ATP hydrolysis. Error bars indicate standard deviation (3 biological replicates). Statistics; unpaired t-test (***p <0.001).

Supplementary Table 1. *Drosophila* genotypes used in this study.

**Genotypes**	**Source**
w[-]; squ^{PP32}*/CyO; TM3, Sb/ TM6, Tb*	Gift from (Pane et al., 2007)
w[-]; squ^{HE47}*/CyO; TM3, Sb/ TM6, Tb*	Gift from (Pane et al., 2007)
w[-];Pin/CyO; spn-E$^{7\text{-}4}$*/TM3, Sb*	This study
w[-]; tej$^{48\text{-}5}$*/CyO*	Patil and Kai, 2010
w[-]; Df(2R)Exel7131/CyO (Df stock for *tej* mutant)	BL#7876
w[-]; vas$^{4\text{-}2}$*/CyO*	This study
w[-]; PBac{WH}krimp^{f06583}*/CyO*	BL #18990<br>(Lim and Kai, 2007)
w[-]; Df(2R)Exel6063/CyO (Df stock for *krimp* mutant)	BL#7545
w[-]; ago3^{g1}*/TM3,Sb*	VDRC#313531
w[-]; ago3^{g2}*/TM3,Sb*	VDRC#313532
w[-]; squ^{HE47}*/CyO;* native-promoter-GFP-3xFlag-Squ^{WT}/TM3, Sb	This study
w[-]; squ^{HE47}*/CyO;* native-promoter-GFP-3xFlag-Squ^{4A}/TM3, Sb	This study
w[-]; Pin/CyO; spn-E$^{7\text{-}4}$*,* native-promoter-GFP-3xFlag-Squ^{WT}*/TM3, Sb*	This study
w[-]; Pin/CyO; spn-E$^{7\text{-}4}$*,* native-promoter-GFP-3xFlag-Squ^{4A}*/TM3, Sb*	This study
w[-]; squ^{PP32}*, NGT40*-Gal4/ *CyO; nos*-Gal4 VP16	This study
w[-]; squ^{HE47}*/CyO;* UASp-3xFlag.AGO3.WT / *TM3, Sb*	This study
w[-]; squ^{PP32}*/CyO;* Spn-E-mk2	This study

Supplementary Table 2. List of primers used for qRT-PCR in this study.

Primer label	Primer sequence	Source
**Rp49_Fw**	ATGACCATCCGCCCAGCATAC	(Lin et al., 2023)
**Rp49_Rv**	CTGCATGAGCAGGACCTCCAG	(Lin et al., 2023)
**Tubulin_Fw**	GGTAACCGTCGAAATCAGTGTT	(Lin et al., 2023)
**Tubulin_Rv**	TGGCTTTTCTGCTATACGTGTC	(Lin et al., 2023)
**38C_Fw**	TCCGTGACGGTTTAGCCCA	(Lin et al., 2023)
**38C_Rv**	AGGTTTCAAACCTTCCAG	(Lin et al., 2023)
**42AB #1_Fw**	CGTCCCAGCCTACCTAGTCA	(ElMaghraby et al., 2019)
**42AB #1_Rv**	ACTTCCCGGTGAAGACTCCT	(ElMaghraby et al., 2019)
**42AB #2_Fw**	CGCTGTTGAAAGCAAATTGA	(ElMaghraby et al., 2019)
**42AB #2_Rv**	GAGACCTTCGCTCCAGTGTC	(ElMaghraby et al., 2019)
**blood_Fw**	CCAACAAAGAGGCAAGACCG	(Zhang et al., 2021)
**blood_Rv**	TCGAGCTGCTTACGCATACTGTC	(Zhang et al., 2021)
**HMS-Beagle_Fw**	CTGTTGACCCTTATTCGCCG	This study
**HMS-Beagle_Rv**	CCGAGAATCCGAGCACTTTG	This study
**Burdock_Fw**	AGGGAAATATTTGGCCATCC	(Czech et al., 2013)
**Burdock_Rv**	TTTTGGCCCTGTAAACCTTG	(Czech et al., 2013)
**42-RT-Sense**	CGAAGCCTTAGATCTCGCTCC	(Klattenhoff et al., 2009)
**42-RT-Antisense**	ACATCAGGAACACAGCGAGGTG	(Klattenhoff et al., 2009)
